# Supplementary material for: ESG growth catalyst: China’s Central Bank collateral framework expansion
Source: PLoS One. 2025 May 19;20(5):e0323726. doi: 10.1371/journal.pone.0323726 (PMC12088038; doi:10.1371/journal.pone.0323726)
Supplement: Attachment — Submitted filename: Response to Reviewers.docx [file pone.0323726.s003.docx]

Dear Editors and Reviewers：

We feel great thanks for your professional review work on our article. As you are concerned, there are several problems that need to be addressed. According to your nice suggestion, we have made extensive corrections to our previous manuscript, the detailed corrections are listed below.

Responses to editorial suggestions

Reply to Comment 1:

We have modified the layout of the manuscript according to the template provided by the journal. If the format needs to be modified, I will actively cooperate.

Reply to Comment 2:

We have stated in the revised coverletter that funders played a role in the study as "funders were not involved in the study design, data collection and analysis, decision to publish, or preparation of the manuscript."

Reply to Comment 3:

We support the sharing of data because it facilitates the dissemination of knowledge. However, since most of our research data comes from the WIND database (a commercial database, distributing it without purchasing its rights would violate copyright). Therefore, our study cannot provide data directly, but relevant readers can reproduce the study by obtaining data from the data sources provided in our study.

Reply to Comment 4:

We have checked the guidelines for providing orcid. We originally designated two corresponding authors, but only one corresponding author was reported in the file exported by the submission system. We have contacted the editorial office, but the response from the editorial office is that "another corresponding author (second author) will also receive an email confirming orcid". After repeated checks, only the first author was the corresponding author in the submission system, and the second author did not receive the email to verify orcid. Since the communication is the responsibility of the second author, we want to be clear about the orientation of each person's task. We are very sorry that we have caused you extra work.

Replies to Comment 5:

We have added headings for Figures 1, 2A, 3, 4 and 5

Reply to Reviewer 1's suggestion:

Comment 1. The Introduction needs to be rearranged, and more work is needed to strengthen the theoretical basis. Additionally, how this study bridges the research gaps should be elucidated. The following paper can be a good example to help you improve your paper (Does firm‐level exposure to climate change influence inward foreign direct investment? Revealing the moderating role of ESG performance. Corporate Social Responsibility and Environmental Management. https://doi.org/10.1002/csr.2917).

Our response: First of all, thank you very much for reviewing our work. We think this is a good suggestion. In the reference (Does firm‐level exposure to climate change influence inward foreign direct investment? Revealing the moderating role of ESG performance. Corporate Social Responsibility and After Environmental Management. https://doi.org/10.1002/csr.2917) this paper, we use more literature reorganize the introduction. In addition, we supplement the limitations of the existing research in the introduction, and thus illustrate how our study fills the research gap.

Comment 2. More existing literature should be presented in the introduction. The following paper can be a good example to help you improve your paper (Effect of ESG Performance on Corporate Green Technology Innovation: The Mediating Role of Financial Constraints and Digital Transformation. The Singapore Economic Review, 1-26.).
Our response: We sincerely thank you for your valuable comments. In reference to what you mentioned (Effect of ESG Performance on Corporate Green Technology Innovation: The Mediating Role of Financial Constraints and Digital Transformation. The Singapore Economic Review, 1-26.) After this paper, We have added more literature to the introduction of the revision as evidence. In addition, we cite this paper as an argument in the process of demonstrating the role of enterprises in green technology innovation as a mechanism.

Comment 3. Also, please add the structure of this study at the end of the introduction.
Our response: Thank you for your reminder. We have supplemented the description of the various parts of this study in the last paragraph of the introduction.

Comment 4. The manuscript falls short in terms of theoretical development. What theories can be used as the theoretical basis for your research topic?

Our response: This is a very valuable suggestion and we take it very seriously. After repeatedly consulting relevant literature and books, we supplemented the scarcity theory and information asymmetry theory in Part 2.2 to put forward the hypothesis of our research.

Comment 5. The arguments proposed for Hypotheses 1, 2a and 2b are insufficient to support the proposition of these hypotheses. The authors should address the arguments for each hypothesis separately. Also, Hypothesis 3c should be supported by more existing literature.
Our response: Thank you for pointing this out. Hypothesis 1, 2a, and 2b are indeed inadequate. Therefore, we add more literature as evidence in hypothesis 1, 2a and 2b respectively, and pay more attention to the logical relationship in the discussion. In addition, as to the suggestion that hypothesis 3c needs more arguments, we propose hypothesis 3c based on the perspective of industry spillover and external supervision, and add more literature as arguments.

Comment 6. The theoretical model should be presented in section 2.

Our response: This is a very good suggestion, but because we are very weak in this area, we can not build a suitable mathematical model to carry out theoretical analysis.

Comment 7. The authors should add the section conclusion.

Our response: Thanks for your suggestion, we have added a conclusion section to Section 5 of our study.

Comment 8. The language of this paper is very bad and needs help from native speakers.

Our response: Thanks for your suggestion We feel sorry for our poor writings, however, we do invite a friend of us who is a native English speaker to help polish our article. And we hope the revised manuscript could be acceptable for you.

Reply to Reviewer 2's suggestion:

1. The abstract should clearly highlight the major findings of the study
Our response: First of all, thank you very much for reviewing our work. This is very good advice. Therefore, we highlight the keyword "research results show" in the abstract, and the impact of EPCF on enterprise ESG performance is expressed numerically, so that readers can understand our research more intuitively.

2. Explanation about the contribution of the paper needs to be improved.

Our response: Thank you very much for your suggestion. We have rewritten the marginal contribution of our study in the revised citation section

3. While developing the hypotheses, the author(s) did not provide proper arguments and synthesize the literature. Generally, the literature review has not been locally and extensively discussed with many unexpected hypotheses

Our response: Thank you for your advice. We have added a lot of literature as evidence and restated our hypothesis based on scarcity theory and information asymmetry theory.

4. There is a need for the authors to clearly explain the reasons for including green innovation of corporations in the paper.

Our response: This proposal is well worth our adoption. The inclusion of corporate green innovation in the study not only helps to reveal the mechanism of EPCF policy's impact on corporate ESG performance, but also provides more targeted suggestions for policy makers. We have provided relevant arguments and arguments for considering corporate green innovation in the revised version of the literature Review section 2.2 Financing constraints and the mechanism role of green innovation, and described the process of its mechanism role.

5. The authors need to discuss and justify the period and the sample they used in the methodology

Our response: This suggestion helps to improve the rigor of our research. We have supplemented the discussion on the study period and sample rationality in section 3.3sample selection and data sources.

6. What is the meaning of 5 Discussion in the paper? Is it written in the right place? Please, check

Our response: Thank you for your valuable advice. We have split chapter 5 into two chapters. They are 5.conclusion and recommendations and 6.Research limitations and outlook.

7. There are many typographical errors in the manuscript for instance; (in the abstract ‘be-tween’) (first paragraph of introduction (chain ++management). There is a need for a serious proofreading of the paper

Our response: Thank you very much for pointing this out. We apologize for these oversights and promise not to repeat them in future submissions. Thank you again for your careful inspection. Sincerely,

Xupei Wang

Xupeiwang1021@126.com
